# The Nordic maintenance care program – case management of chiropractic patients with low back pain: A survey of Swedish chiropractors

**DOI:** 10.1186/1746-1340-16-6

**Published:** 2008-06-18

**Authors:** Iben Axén, Annika Rosenbaum, Andreas Eklund, Laszlo Halasz, Kristian Jørgensen, Peter W Lövgren, Fredrik Lange, Charlotte Leboeuf-Yde

**Affiliations:** 1 Private practice and the Karolinska Institute, Stockholm, Sweden; 2 Private practice, Linköping, Sweden; 3 Private practice, Stockholm, Sweden; 4 Private practice, Lund, Sweden; 5 Research Professor, Nordic Institute for Chiropractic and Clinical Biomechanics, Part of Clinical Locomotion Science, University of Southern Denmark, Denmark[

## Abstract

**Background:**

Chiropractic treatment for low back pain (LBP) can often be divided into two phases: Initial treatment of the problem to attempt to remove pain and bring it back into its pre-clinical or maximum improvement status, and "maintenance care", during which it is attempted to maintain this status. Although the use of chiropractic maintenance care has been described and discussed in the literature, there is no information as to its precise indications. The objective of this study is to investigate if there is agreement among Swedish chiropractors on the overall patient management for various types of LBP-scenarios, with a special emphasis on maintenance care.

**Method:**

The design was a mailed questionnaire survey. Members of the Swedish Chiropractors' Association, who were participants in previous practice-based research, were sent a closed-end questionnaire consisting of nine case scenarios and six clinical management alternatives and the possibility to create one's own alternative, resulting in a "nine-by-seven" table. The research team defined its own pre hoc choice of "clinically logical" answers based on the team's clinical experience. The frequency of findings was compared to the suggestions of the research team.

**Results:**

Replies were received from 59 (60%) of the 99 persons who were invited to take part in the study. A pattern of self-reported clinical management strategies emerged, largely corresponding to the "clinically logical" answers suggested by the research team. In general, patients of concern would be referred out for a second opinion, cases with early recovery and without a history of previous low back pain would be quickly closed, and cases with quick recovery and a history of recurring events would be considered for maintenance care. However, also other management patterns were noted, in particular in the direction of maintenance care.

**Conclusion:**

To a reasonable extent, Swedish chiropractors participating in this survey appear to agree on the clinical management for different cases of LBP.

## Background

According to experience, chiropractic treatment can often be divided into two phases: Initial treatment of the problem to attempt to bring it back into its pre-clinical or maximum improvement status, and "maintenance care", during which it is attempted to maintain this status. The first definition of maintenance care that we could find in the literature was provided by Breen in 1977 [1]: "...treatment, either scheduled or elective, which occurred after optimum recorded benefit was reached..." and the second definition that we could locate was provided by Mitchell in 1980 [2]: "A regimen designed to provide for the patient's continued well-being or for maintaining the optimum state of health while minimizing recurrences of the clinical status". In "Advances in Chiropractic" from 1996, the word "maintenance care" is defined as follows: "Appropriate treatment directed toward maintaining optimal body function. This is treatment of the symptomatic patient who has reached pre-clinical status or maximum medical improvement, where condition is resolved or stable" [3]. In other words, maintenance care can be described as both an attempt at secondary prevention (preventing further events from occurring) and tertiary prevention (maintaining an incurable condition at an acceptable level).

According to the literature, spinal manipulative therapy is an important aspect of the maintenance care approach [4-7], but also other aspects could be included, such as advice, information, and counselling [4,6,8] even in relation to general health promotion [9]. However, the indications for maintenance care [10,11] and clear descriptions of preventive treatment for specific types of conditions are not found in the literature. Also, general concepts of how to proceed over time with this type of patient are lacking, and the therapeutic value of maintenance care has not been tested, with the exception of a promising pilot study [12].

Despite this lack of scientific support, it was shown that American chiropractors share a common understanding about the purpose and composition of maintenance care and that they recommend it to the majority of their patients [4]. However, it is not known if there is a general or uniform management culture among chiropractors. In relation to the decision to treat a patient with spinal manipulative therapy, there are various schools of thought within the chiropractic profession. Some chiropractors are guided by both their own clinical findings and the patients' symptoms whereas others largely disregard the patients' symptoms, as described in a guideline on the vertebral subluxation in chiropractic practice: "Because the duration of care is being considered relative to the correction of vertebral subluxation, it is independent of clinical manifestations of specific dysfunctions, diseases, or syndromes." [13]. Maintenance care would therefore probably be undertaken differently for these two groups; the former group using "symptom-guided maintenance care" whereas the approach of the second group would be "clinical findings-guided maintenance care".

We were interested in finding out whether there is agreement among chiropractors regarding their management for various types of patient groups. In particular, we wanted to find out when chiropractors would recommend maintenance care.

Many patients who visit chiropractors suffer from low back pain (LBP). It was therefore logical to start this work on chiropractic patients with LBP. The results from this study may create a base from which further research into maintenance care can be conducted with the ultimate aim to investigate its clinical usefulness. Several such projects are presently underway.

## Method

### Study Procedure

A questionnaire was designed describing various LBP-scenarios at the end of the initial more intensive treatment period, when a decision about maintenance care would be made. For each scenario the chiropractors could choose from a number of management strategies, including the option of maintenance care. In other words, the chiropractors were to match each scenario with the management strategy of his/her choice.

The questionnaire was distributed to a group of Swedish chiropractors in the spring of 2006. Replies were returned in pre-printed and pre-stamped envelopes.

### The Research Team

The research team consisted of a group of seven chiropractors, having obtained their chiropractic degree in the US, Australia, UK or Denmark with a clinical experience ranging from 4 to 25 years. This group was supervised by a professional chiropractic researcher (CLY).

### Design and Tests of the Questionnaire

A questionnaire was designed in English by the research team, with the purpose of describing a range of clinical scenarios and finding out which management strategies chiropractors would prefer to use for these scenarios. In addition, practitioners were asked if they use "maintenance care" in their practice and if so, the proportion of such patients on the day of the study. Similarly to a previous study [4], we purposefully did not include a definition of maintenance care or descriptions of what therapies might be included, in our instructions to the participants. In fact, we informed them that the reason for the study was a lack of clarity on the subject. The questionnaire is included in Additional File 1.

In order to make the questionnaire as brief and clear as possible, an uncomplicated case was used as a basis for nine possible outcomes that were briefly described ("scenarios"). The basic facts for this hypothetical patient were: "A 40-year old man who consults you for Low Back Pain with no additional spinal or musculoskeletal problems, and with no other health problems. His X-rays are normal for his age. There are no "red flags". Clearly, X-rays would not be indicated in real life [14] but this information was included to emphasise that there was no obvious spinal pathology present.

The nine different scenarios were described in relation to outcome after the initial treatment period. These outcomes had to be described in overall terms as all chiropractors do not use the same methods of evaluation of their patients' progress. Outcome was described on the basis of "pain" because it is highly relevant for both patients and chiropractors during the initial treatment of LBP, regardless how it is measured. Treatment regimes also differ between chiropractors, making it necessary also here to provide simplistic situations in relation to number of treatments and duration of treatment.

After each of these nine scenarios there were six possible management strategies, preceded by the question: "What would you recommend?" It was also possible to suggest one's own management strategy alternative. For ease of reporting, brief terms will be used in this report to describe these management strategies (Additional File 2).

The contents and wording of the questionnaire were pilot tested once by a small number of chiropractors, adjusted in response to their comments and tested once more on three chiropractors with a research background. They detected some logical errors in the description of the scenarios and suggested some changes to the management strategies, which resulted in further improvements to the contents, wording and lay-out of the questionnaire.

### The Clinical Significance of the Nine Scenarios in the Questionnaire

The nine scenarios were constructed in such a way as to include cases that went from uncomplicated to more difficult, including scenarios with no past history of LBP, those with intermittent LBP over the past year, and those with several similar events over the past year. The research team had anticipated that patients with fast recovery and no previous history of LBP would be quickly completed, whereas those who responded well to treatment and who had a long-lasting history of LBP would be candidates for maintenance care. We also assumed that patients with a more complicated clinical course during the initial treatment period would be submitted to a change in treatment strategy, or referred out for additional therapy (such as training), and that cases of concern would be referred out for a second opinion. Specifically, we expected that a prerequisite for maintenance care was that the patient experienced considerable improvement.

In this study we defined improvement in relation to percent improvement of pain. Our scenarios included the following possibilities for pain outcome: "completely gone" (i.e. 100% better), "80% better", "50% better", and "20% better". The difference between 50% and 20% was deliberately made large in order to indicate that the 20% improvement was clinically unsatisfactory. Please, see Additional File 3 for the clinical reasoning of the research team and a description of their preferred management strategy for each scenario.

### Participants

Chiropractors were invited to the study if they were members of the Swedish Chiropractors' Association, "Legitimerade Kiropraktorers Riksorganisation" (LKR), and if they had previously actively participated in practice-based research project. The LKR, at the time of the study, consisted of 160 members.

Over the past years, also locally trained so-called chiropractors have obtained legal recognition in Sweden. However, because their education, after inspection of their school, was not approved by the governmental body (the Swedish Board of Education) [15], and because their school also has failed to become approved by the European Council on Chiropractic Education (ECCE), they are not allowed membership in the LKR, nor can their own association obtain membership in the European Chiropractors' Union. In other words, although they call themselves chiropractors, they cannot be considered typical of the European chiropractic profession. Therefore, that group of chiropractors was not invited to participate in this study.

### Analysis and Reporting of Data

The data were analyzed manually by the members of the research team. The percentage of responses (A, B, C etc.) for each hypothetical scenario was calculated. Explanations provided under "none of the above. Please explain..." (G) were scrutinized for contents and recoded into the correct box, if possible, or else left under G. An extra response possibility was added consisting of "multiple answers". Thereafter, the number of times that each strategy was selected for each scenario was calculated. Finally, the proportion of so-called "maintenance care" patients on the day of the survey was calculated for each practitioner to make it possible to estimate the mean and median proportion of maintenance care patients in the entire group of responders.

### Ethics

All participants were anonymous and the questionnaire contained no information that could identify the participants. Studies of this type do not require permission from the local ethics committee.

## Results

Fifty-nine chiropractors of the 99 potential participants (60%) returned their questionnaire. The proportion of patients who were reported to have been seen under a "maintenance care"-scheme, on the day of the survey, ranged between 2% and 95% (mean 28.6 and median 20).

### Choice of management strategy – summary of findings

As can be seen in Table 1, the largest prevalence of preferred management strategy for each case scenario ranged from 25% to 59%.

**Table 1:** How 59 Swedish chiropractors would choose their continued case management strategies (A-G) in nine hypothetical case scenarios of LBP (%).

**Strategies**	**A **	**B **	**C **	**D **	**E **	**F **	**G **	**Several replies**	**Don't know**
	**2^nd ^opinion**	**Quick-fix**	**Try again**	**Ext. help – keep in touch**	**Symptom-guided maintenance care**	**Clinical findings-guided maintenance care**	**Other**		
**The 9 case scenarios**									
1	0	54*	2	0	20	19	2	2	2
2	0	14	3	0	44*	30	2	2	5
3	20	0	42*	12	8	10	3	0	3
4	0	7	3	3	46*	34*	2	0	5
5	14	0	25*	17*	10	24	3	2	5
6	5	0	37*	29*	7	10	3	2	7
7	15	2	32*	15*	12	8	5	2	8
8	59*	0	14	14	0	0	3	2	8
9	59*	0	8	19	0	0	3	2	8

*TOTAL NUMBER OF REPLIES*	*102*	*45*	*99*	*64*	*87*	*80*	*16*	*7*	*31*

The largest estimate for each case scenario has been highlighted. Descriptions of the different case scenarios and management strategies are found in App. 2 and 3.

* denotes the pre-hoc choices of the research team.

A closer look at the various preferred management strategies for the nine case scenarios told the following story: "Second opinion" would be recommended for the patient who got gradually worse (scenario 8) and for another patient, who did not improve and had signs of other problems (scenario 9). The "quick fix"-option was selected for scenario 1, the patient who improved quickly, was uncomplicated and had no past LBP-history. "Try again" was considered particularly relevant for scenarios 3,5,6 and 7; all patients who failed to improve quickly and well but did not appear to have any warning signs. "Symptom-guided maintenance care" was predominantly selected for scenarios 2 and 4. Case 2 was described as a patient without past LBP, who recovered quickly but feared future problems and case 4 made good recovery but had a history of recurrent problems. "Clinical-findings guided maintenance care" and "External help – keep in touch" were never first choice. The preferred pattern of management strategies was largely in agreement with the pre hoc choices made by the research team.

Two of the strategies could be classified as "maintenance care" (symptom-guided maintenance care" and "clinical-findings guided maintenance care"). When combined, some type of maintenance care achieved the second highest frequency of responses also for scenarios 1 and 5, whereas none of the respondents suggested this type of strategy for cases 8 and 9, who most thought were suitable for "second opinion". If the two types of maintenance care were combined, between 20% and 80% of the respondents would recommend maintenance care for all the scenarios but 8 and 9.

## Discussion

### Discussion of findings

Among the Swedish chiropractors who participated in this survey, a distinct pattern was found, in relation to the management strategies that they would choose for different types of LBP-scenarios. This pattern corresponded to that which the research team, arbitrarily, considered to be logical and responsible.

However, also other patterns were apparent, sometimes favouring a prolonged management program, either symptom-guided or clinical-findings guided, indicating that some chiropractors have high expectations of "a happy ending" to many clinical conditions. The "quick-fix" alternative was not often selected but, then, only cases 1, 2 and 4 were described as completely improved, and therefore the only ones obviously suitable to be considered for closure.

Nevertheless, it is reassuring to see that for the potentially serious cases 8 and 9, the most common strategy would have been referral for "second opinion" and that, for these, none of the participants would have considered any type of maintenance care.

Another interesting finding is that some chiropractors seem to fail to grasp the concept of clinically significant improvement. For example, in case 5, an acute event of LBP of one week's duration that is only 20% better after one month and six visits does not appear to be the suitable recipient for clinical findings-guided maintenance care. Nonetheless, this approach was the second most commonly selected strategy for this case, and if both types of maintenance care were considered together, this approach was, in fact, the most preferred choice. It has been shown that patients need to experience more substantial reduction of pain before it can be considered clinically significant [16]. In fact, mere diurnal fluctuations and measurement errors could probably account for an improvement of 20%. In our opinion, maintenance care should only be considered in patients who have responded well to the initial treatment and only in patients who are likely to experience frequent or long-lasting problems in the future. Admittedly though, this is only our humble opinion, and the true indications for maintenance care remain to be studied.

According to a previous study of osteopaths, chiropractors and physiotherapists a subgroup of clinicians will provide prolonged treatment also for patients with LBP, who do not recover. The reasons for this seemed to be linked with a scope of care, which encompasses more than the immediate symptomatic relief [17]. Obviously, the different aspects of clinical reasoning need to be studied in order to understand various choices of management strategies.

### Methodological considerations and comparisons with other studies

Whether these results can be trusted or not and whether they can be generalized or not, depends on several factors. First, the chiropractors who were invited to participate in the study would best be described as a convenience sample, as they consisted of colleagues who had participated in previous studies. It is possible that participants in research projects are more academically inclined than others, which obviously may have an impact on their practice pattern and the rationale for how they practice.

Despite this pre-selection of participants, the response rate was rather low (60%). In comparison, the response rate was 44% in a North American questionnaire survey on maintenance practice patterns [4]. This was anticipated in our study because this survey was distributed together with material for a larger study (unrelated to maintenance care), which included a somewhat complicated study procedure. It is our experience that chiropractors will be fairly compliant in studies requiring a minimum of activities from their side and which require no more than 1–2 minutes per patient. Those who are compliant in more complex studies are probably likely to be more interested in research, to have secretarial assistance, or – perhaps – to be less busy. In what way this affects the results, is unknown. It would therefore be necessary to verify these findings in other study populations. Such studies are in process.

In previous studies, the prevalence estimates of the use of maintenance care were 39% in a file search among British chiropractors in 1973–4 [1], and 14% in a Norwegian multicenter clinical outcome study [18].

The results, however, are not really comparable. The British study is more than 30 years old and included all types of patients and the Norwegian study had information from chiropractors' own file search regarding the participating patients, who all had persistent LBP. Obviously, it is not possible to judge the external validity of our study by comparing our percentage of maintenance care patients to those of previous studies of similar study populations.

Having obtained the study subjects, it is also important that they understand the questionnaire and respond to it in a manner that corresponds to their clinical behaviour. Our participants had previously participated in several practice-based research projects and were experienced with questionnaires. The pilot study helped remove the obviously unsuitable questions and made the questionnaire easier to read and to answer. However, because the case scenarios were very simplistic, there would always be room for individualized interpretations that could affect the study results. Some of the respondents failed to answer all questions, but there were only between 1 and 5 "don't know" responses for the various cases, indicating that the questionnaire was relatively user-friendly.

The issue of maintenance care is, by some, considered to be a sensitive issue. It was therefore important that the questionnaire was returned anonymously and we therefore assume that the respondents provided honest answers to the questions, even if these were considered "politically incorrect".

The choice of clinical management programs may depend on the educational background. Swedish chiropractors are mainly educated in North America or UK. Only a few are educated at the University of Southern Denmark and all the included chiropractors had been working mainly in Sweden or other European countries. It is therefore not certain that the results from this study are typical for other groups of chiropractors.

It is also important that the choice of responses cover most possible management possibilities. Some chiropractors claimed that they had an "other" alternative to those proposed in the questionnaire. However, when their responses were scrutinized, there remained only 16 replies that could not easily be placed under one of the pre-printed alternatives. Most of these consisted of general discussions of patient care and failed to address the question to be answered. No "new" alternatives were detected from the "other" alternative, indicating that our choice of management strategies was satisfactory. In our experience, it is not uncommon that clinicians claim that it is impossible to fit their answers into predefined boxes, such as describing a treatment program based on theoretical cases, because they claim that each case is unique. Nevertheless, this study showed that, at least, this group of chiropractors was able to do so to a large extent.

## Conclusion

Among those chiropractors who participated in this survey, a clinical management strategy pattern emerged for different cases of LBP. However, there were also subgroups of chiropractors with different practice cultures, sometimes favouring a maintenance care program. The rationale for their clinical decisions needs to be further elucidated, and the results of this study need to be verified in other study populations with a variety of study designs.

## Competing interests

The authors declare that they have no competing interests.

## Authors' contributions

IA was responsible for the design of the study, supervision of data collection, the analysis of data and the manuscript preparation, AR, AE, LH, KJ, FL and PWL were involved in the design, supervision of data collection and the analysis of data, CLY was supervising the study process and was involved in the manuscript preparation. All authors revised and approved the final manuscript.

## Supplementary Material

Additional file 1 A Questionnaire mailed to 99 Swedish chiropractors asking them to match nine case scenarios with six specific management strategies. Click here for file

Additional file 2 A description of the six specific management strategies for patients with low back pain receiving chiropractic care, from which the participants in the survey could select one for each of nine scenarios. Note: A brief description for each strategy is included in brackets, used in the report. Click here for file

Additional file 3 A description of nine scenarios (cases 1 – 9), together with the clinical reasoning of the research team, and a description of their preferred management strategy for each scenario (not included in the questionnaire). Click here for file
