# Supplementary material for: The Nordic maintenance care program – case management of chiropractic patients with low back pain: A survey of Swedish chiropractors
Source: Chiropr Osteopat. 2008 Jun 18;16:6. doi: 10.1186/1746-1340-16-6 (PMC2442107; doi:10.1186/1746-1340-16-6)
Supplement: Additional file 1 — A Questionnaire mailed to 99 Swedish chiropractors asking them to match nine case scenarios with six specific management strategies. [file 1746-1340-16-6-S1.doc]

A Questionnaire mailed to 99 Swedish chiropractors asking them to match nine case scenarios with six specific management strategies.

*Dear Colleague, CODE __ __*

*Our next research area will be about maintenance care. First, we need to find out what we really mean by “maintenance care”, because no clear definition and description exists in the chiropractic literature. For this reason we very much need your help. We want to find out what we, the professional chiropractors in Sweden, mean by maintenance care and how it is used in everyday practice.*

*We are therefore asking you to fill out this questionnaire. When we have received your response, the code will be removed from the questionnaire, and all analyses and final reporting will be on an anonymous basis.*

*First, please answer the following questions by encircling your response:*

**Do you use maintenance care in your practice? Yes No**

**If yes, in your last full working day, how many maintenance care patients did you have**

**of your total that day? ----- maintenance care out of -------total.**

**_________________________________________________________________________________**

*Please, read the following cases and, for each case, give the answer that you consider fits best with the decision you would make in a clinical setting.*

*We have selected an imaginary patient, as described in the box below. Then, different scenarios for this patient are outlined, and you are asked to select ONE of several clinical solutions (A, B, C etc.) as listed in bold letters below.*

*You can choose between the following possibilities for each of the cases presented below:*

1. **I would refer the patient to another health care practitioner for a second opinion.**
2. **I would advise the patient to seek additional treatment whilst following the patient.**
3. **I would tell the patient that the treatment is completed but that he is welcome to make a new appointment if the problem returns.**
4. **I would not consider the treatment to be fully completed and would try a few more treatments and perhaps change my treatment strategy, until I am sure that I cannot do any more.**
5. **I would follow this patient for a while, attempting to prolong the time period between visits until either the patient is asymptomatic or until we have found a suitable time lapse between check-ups to keep the patient symptomfree.**
6. **I would recommend that the patient continues with regular visits, as long as clinical findings indicate treatment (eg spinal dysfunction/subluxation) even if the patient is symptomfree.**
7. **Neither of the above. (Please explain at the back of the page in legible handwriting)**

These are the basic facts for our hypothetical patient:

A 40-year old man who consults you for Low Back Pain with no additional spinal or musculoskeletal problems, and with no other health problems.

His X-rays are normal for his age.

There are no “red flags”.

*The case above could proceed in the* ***following 9 ways*** *described on the next page.*

*Please encircle the letter that corresponds best to your clinical judgement in each of the cases.*

CODE __ __

An acute attack of LBP of 2 days´ duration and no previous history of LBP. The pain is completely gone after 2 visits. The patient seems to be an uncomplicated person and capable to look after himself and his back.

**What would you recommend? A B C D E F (G )**

1.

An acute attack of LBP of 2 days´duration and no previous history of LBP. The pain is completely gone after 2 visits. The patient is very worried that the pain will come back again. The patient asks if he could come back regularly to make sure this will not happen.

**What would you recommend? A B C D E F (G )**

2.

An acute attack of LBP of 2 days´ duration and no previous history of LBP. The pain is about 20% better after 6 visits.

**What would you recommend? A B C D E F (G )**

3.

An acute attack of LBP of 1 week´s duration. The patient has had several similar attacks over the past 12 months. The pain is completely gone after 2 weeks of treatment.

**What would you recommend? A B C D E F (G)**

**g )**

4.

An acute attack of LBP of 1 week´s duration. The patient has had several similar attacks over the past 12 months, but the pain pattern has varied over the treatment period and now, after six visits, the pain is 20% better.

**What would you recommend? A B C D E F (G )**

**Choose: A B C D E F G**

5.

The patient has had LBP intermittently over the past year. After the 2^nd^ visit, the pain was 50% better but today, after six visits there has been no further change.

**What would you recommend? A B C D E F (G )**

6.

The patient has had LBP intermittently over the past year. After 6 visits, the pain was 80% better, but after a further two treatments the last month, the problem has gradually got a bit worse. **What would you recommend? A B C D E F (G)**

7.

The patient has had LBP intermittently over the past year. After the 2^nd^ visit the pain was 20% better, but today, after 6 visits and over the past month, the patient has got gradually worse.

**What would you recommend? A B C D E F (G )**

8.

The patient has had LBP intermittently over the past year. After 6 visits the pain is 20% better. The symptoms come and go for no apparent reason. The patient appears tired and moody.

**What would you recommend? A B C D E F (G)**

9.
