# Supplementary material for: The Nordic maintenance care program – case management of chiropractic patients with low back pain: A survey of Swedish chiropractors
Source: Chiropr Osteopat. 2008 Jun 18;16:6. doi: 10.1186/1746-1340-16-6 (PMC2442107; doi:10.1186/1746-1340-16-6)
Supplement: Additional file 2 — A description of the six specific management strategies for patients with low back pain receiving chiropractic care, from which the participants in the survey could select one for each of nine scenarios. Note: A brief description for each strategy is included in brackets, used in the report. [file 1746-1340-16-6-S2.doc]

A description of the six specific management strategies for patients with low back pain receiving chiropractic care, from which the participants in the survey could select one for each of nine scenarios. A brief description for each strategy is included in brackets, used in the report.

1. I would refer the patient to another health care practitioner for a second opinion. (“second opinion”)
2. I would tell the patient that the treatment is completed but that he is welcome to make a new appointment if the problem returns. (“quick-fix”)
3. I would not consider the treatment to be fully completed and would try a few more treatments and perhaps change my treatment strategy, until I am sure that I cannot do any more. (“try again”)
4. I would advise the patient to seek additional treatment whilst following the patient. (“external help – keep in touch”)
5. I would follow this patient for a while, attempting to prolong the time period between visits until either the patient is asymptomatic or until we have found a suitable time lapse between check-ups to keep the patient symptoms free. (“symptom-guided maintenance care”)
6. I would recommend that the patient continues with regular visits regardless of symptoms, as long as clinical findings indicate treatment (e.g. spinal dysfunction/subluxation). (“clinical findings-guided maintenance care”)
