# Supplementary material for: The Nordic maintenance care program – case management of chiropractic patients with low back pain: A survey of Swedish chiropractors
Source: Chiropr Osteopat. 2008 Jun 18;16:6. doi: 10.1186/1746-1340-16-6 (PMC2442107; doi:10.1186/1746-1340-16-6)
Supplement: Additional file 3 — A description of nine scenarios (cases 1 – 9), together with the clinical reasoning of the research team, and a description of their preferred management strategy for each scenario (not included in the questionnaire). [file 1746-1340-16-6-S3.doc]

A description of nine scenarios (cases 1 – 9), together with the clinical reasoning of the research team, and a description of their preferred management strategy for each scenario (not included in the questionnaire).

*Case 1. An acute attack of LBP of 2 days’ duration and no previous history of LBP. The pain is completely gone after 2 visits. The patient seems to be an uncomplicated person and capable to look after himself and his back.*

According to the research team, this case indicates a person without a background of persistent or recurrent LBP, with a quick recovery and a psychological profile that indicates a good prognosis. The team would have selected strategy B (“I would tell the patient that the treatment is completed but that he is welcome to make a new appointment if the problem returns”).

*Case 2. An acute attack of LBP of 2 days’ duration and no previous history of LBP. The pain is completely gone after 2 visits. The patient is very worried that the pain will come back again. The patient asks if he could come back regularly to make sure this will not happen.*

The thoughts of the research team were that, ideally, this patient should be dismissed, similarly to the case above (strategy B). However, the psychological profile of this patient needs to be taken into account and he should be provided with a sense of security whilst guided by the chiropractor and gradually weaned off to prevent dependency upon chiropractic treatment. The team therefore selected strategy E, with the intent of using a couple of more visits to improve the patient’s self-confidence.

*Case 3. An acute attack of LBP of 2 days’ duration and no previous history of LBP. The pain is about 20% better after 6 visits.*

This patient is not improving at a level and rate that should be expected. Because the basic case states that there are no red flags, the team decided that this case should be reconsidered and a few more attempts made. The strategy that best suited for this scenario was C.

*Case 4. An acute attack of LBP of 1 week’s duration. The patient has had several similar attacks over the past 12 months. The pain is completely gone after 2 weeks of treatment.*

This is a recurrent problem according to the past history. If the patient considers that the chiropractic treatment shortened the duration of the typical attack, he should simply return as soon as a new problem is felt to commence. Unfortunately, many patients will fail to do so, thinking that the treatment did not help when it starts up again. It might therefore be advantageous to keep an eye on the patient for a while with the intent of finding out if each event of LBP can be quickly and efficiently treated at a “cost-effect “ time interval (strategy E) or if it is possible to prevent further events (strategy F).

*Case 5. An acute attack of LBP of 1 week’s duration. The patient has had several similar attacks over the past 12 months, but the pain pattern has varied over the treatment period and now, after six visits, the pain is 20% better.*

This patient is not improving at a level that should be expected despite the large number of visits, indicating that he may be resistant to the type of treatment that has been provided so far. A change of strategy would be required (strategy C) or if the patient is referred out, it would be relevant to keep in touch to be able to be of support in the continued process (strategy D).

*Case 6. The patient has had LBP intermittently over the past year. After the 2^nd^ visit, the pain was 50% better but today, after six visits there has been no further change.*

This patient may have reached his optimal stage with the present type of treatment and the therapy should, at this stage, either be reconsidered “in-house” or by someone else, indicating strategy C or D.

*Case 7. The patient has had LBP intermittently over the past year. After 6 visits, the pain was 80% better, but after a further two treatments the last month, the problem has gradually got a bit worse.*

The team used the following reasoning: The improvement seen, to date, may have been independent of the treatment and merely an expression of the typical intermittent pain pattern, or the treatment did have an effect but there is something that re-aggravated the condition. The team would, therefore, have reconsidered the case (strategy C) or sent the patient out for an adjunctive approach, such as training, whilst keeping in touch (strategy D).

*Case 8. The patient has had LBP intermittently over the past year. After the 2^nd^ visit the pain was 20% better, but today, after 6 visits and over the past month, the patient has gradually got worse.*

This patient has not really exhibited a positive response to the treatment and is, in fact, getting worse. That the patient is gradually worsening is not a normal pattern. Despite the fact that there are no (obvious) red flags the team would refer the patient for a second opinion (strategy A), because some underlying explanatory condition could have been missed.

*Case 9. The patient has had LBP intermittently over the past year. After 6 visits the pain is 20% better. The symptoms come and go for no apparent reason. The patient appears tired and moody.*

This patient has not improved at all and there is no obvious (biomechanical) explanation for the intermittent pattern. There are no red flags but there is a need to consider if there might not be an underlying depression or some other disease, after all. The team would not hesitate to refer out for a second opinion (strategy A).
